# Protein kinase A type I activates a CRE-element more efficiently than protein kinase A type II regardless of C subunit isoform

**DOI:** 10.1186/1471-2091-12-7

**Published:** 2011-02-08

**Authors:** Øystein Stakkestad, Anja CV Larsen, Anne-Katrine Kvissel, Sissel Eikvar, Sigurd Ørstavik, Bjørn S Skålhegg

**Affiliations:** 1 Department of Nutrition, Institute for Basic Medical Sciences, University of Oslo, Sognsvannsveien 9, P.O. Box 1046 Blindern, N- 0316 OSLO, Norway; 2 Department of Biochemistry, Institute for Basic Medical Sciences, University of Oslo, Sognsvannsveien 9, P.O. Box 1112 Blindern, N- 0317 OSLO, Norway; 3 Department of Pathology, The Norwegian Radium Hospital, Oslo University Hospital, Ullernchausseen 70, N-0310 Oslo, Norway; 4 Department of Oncology, Ullevål Hospital, Oslo University Hospital, Kirkeveien 166, P.O. Box 4950, Nydalen N-0424, OSLO, Norway

## Abstract

**Background:**

Protein kinase A type I (PKAI) and PKAII are expressed in most of the eukaryotic cells examined. PKA is a major receptor for cAMP and specificity is achieved partly through tissue-dependent expression and subcellular localization of subunits with different biochemical properties. In addition posttranslational modifications help fine tune PKA activity, distribution and interaction in the cell. In spite of this the functional significance of two forms of PKA in one cell has not been fully determined. Here we have tested the ability of PKAI and PKAII formed by expression of the regulatory (R) subunits RIα or RIIα in conjunction with Cα1 or Cβ2 to activate a co-transfected luciferace reporter gene, controlled by the cyclic AMP responsive element-binding protein (CREB) *in vivo*.

**Results:**

We show that PKAI when expressed at equal levels as PKAII was significantly (p < 0.01) more efficient in inducing Cre-luciferace activity at saturating concentrations of cAMP. This result was obtained regardless of catalytic subunit identity.

**Conclusion:**

We suggest that differential effects of PKAI and PKAII in inducing Cre-luciferace activity depend on R and not C subunit identity.

## Background

Cyclic 3', 5'-adenosine monophosphate (cAMP) is a key intracellular signaling molecule, which main function is to activate the cAMP-dependent protein kinases (PKA) [1,2]. PKA is a heterotetrameric holoenzyme composed of two regulatory (R) and two catalytic (C) subunits, which is enzymatically inactive in the absence of cAMP. When two molecules of cAMP bind to each of the R subunits [3], the C subunits are released and activated to phosphorylate serine and threonine residues on specific intracellular target proteins [4,5]. Several PKA substrates have been identified of which the synthetic peptide Kemptide [6] and the naturally occurring substrate cAMP responsive element binding protein (CREB) are of the best characterized [7,8]. In primates, four genes encoding the R subunit and four genes encoding the C subunit, have been identified and designated RIα, RIβ, RIIα, RIIβ and Cα, Cβ, Cγ and X-chromosome encoded protein kinase X (PrKX) [9].

Whereas no splice variants for RIβ and RIIβ have been described, RIα is transcribed from at least two different promoters. The first exons of the RIα gene, exon 1a and 1b, give rise to alternatively spliced but identical proteins RIα1a and RIα1b [10]. RIα 1a and 1b mRNAs have been identified in most tissues and are differentially regulated by cAMP [11-13]. In the case of RII, it has been shown that RIIα in the human testis but no other tissues examined, is encoded with an alternative amino-terminal region [14]. No functional consequences of alternative splicing of RI and RII have been reported.

Several splice variants are transcribed from the Cα and the Cβ genes (PRKCA and PRKCB) and include Cα1, CαS, Cβ1, Cβ2, Cβ3 and Cβ4, in addition to an unknown number of abc forms of the Cβ3 and Cβ4 variants [15-20]. The major differences between the various C subunits are introduced through alternative use of exon 1 in the PRKCB and PRKCA genes, respectively [16,21,22]. In the case of Cα1 exon 1-1 encodes an N-terminal stretch of 14 amino acids that have three sites that undergo co- and posttranslational modifications. At the very N-terminus a Gly is located that undergoes myristoylation *in vivo *[23]. C-terminal to the Gly an Asn is located that is partly deamidated *in vivo *leading to Cα1-Asp2 and Cα1-iso(β)Asp2 [24]. The third modification is PKA-autophosphorylation at Ser10 [25-27]. In the case of Cβ2, exon 1-2 encodes an N-terminal stretch of 62 amino acids that does not harbor sites for any of the modifications identified in Cα1. Instead, the Cβ2 N-terminus contains a stretch of hydrophobic amino acids that form an amphiphatic α-helix displayed as a hydrophobic surface [20]. Cα1 and Cβ1 are more than 90% identical at the amino acid level and are ubiquitously expressed. CαS has only been identified in sperm cells [28], Cβ2 is predominantly expressed in lymphoid cells [29,30], and Cβ3 and Cβ4 and their abc variants are mainly expressed in neuronal tissues [15,16].

It is assumed that any known C subunit may associate with RI and RII to form PKAI and PKAII, respectively [9]. This has raised the question of the biological significance of PKAI and II holoenzymes containing various C isoforms within the same cell. Whereas no reports have been published on the functional consequences of holoenzymes formed with various C subunits, it has been demonstrated that several cell types expressing RIα are highly proliferative and may also be associated with malignancy [31-34]. Using a genetic approach it has also been demonstrated that constitutive ablation of RIα but not RIβ is prenatal lethal whereas ablation of the RII variants results in more discrete defects, affecting differentiation of adipose tissue and neural functions[35-37]. The levels of RI and RII as well as tissue- and subcellular expression varies. They also show differential affinities for A-kinase anchoring proteins (AKAP). Furthermore, when determining the structure of the PKA holoenzymes it was found that RI and RII contact the substrate binding site of the C subunit either as a true PKA substrate (RII) or as a pseudosubstrate (RI) due to autophosphorylation of RII but not RI at Ser95 [38,39]. Despite these differences an explanation for biological differences at the cellular level between RI and RII are not fully appreciated [40,41]. However, it should be noted that RII autophosphorylation modulates AKAP-RII interaction in cardiac cells, leading to altered down-stream substrate phosphorylation and Ca^2+ ^dynamics [42].

To investigate biological differences between RI and RII and to demonstrate if such differences are dependent on C subunit identity we formed PKAI and PKAII by co-transfecting 293T cells with either RIα or RIIα together with Cα1 and Cβ2, respectively. This demonstrated that PKAI was superior to PKAII in activating a cAMP responsive element regardless of whether the holoenzyme contained Cα1 or Cβ2. Our results contribute to understand the functional significance of two PKA holoenzymes but not various C subunits expressed in the same cell.

## Results

**Figure 1:**
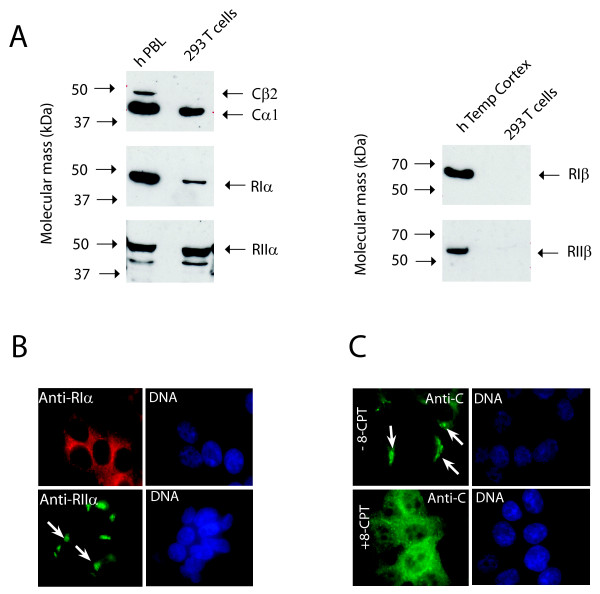
**PKAI (RIαCα1) and PKAII (RIIαCα1) are expressed in 293T cells**. **(A) **Cell extracts of 293T cells (40 μg protein/lane) were analyzed by immunoblotting using a pan-anti-C antibody (upper left panel) and anti-RIα and anti-RIIα (lower left panels). The levels and identities of 293T cell C and R subunit expression were compared to human peripheral blood lymphocytes (hPBL) revealing expression of Cα1, RIα and RIIα. No detectable levels of RIβ and RIIβ were identified when compared to extracts of human temporal cortex (hTempCortex, right panels). (**B**) Immunofluorescence analysis of PKA RI and RII in 293T cells. RIα (Anti-RI, red) is expressed diffusely in the cytosol and RIIα (Anti-RII, green) is expressed in the Golgi-centrosomal area of 293T cells. (**C**) Immunofluorescence analysis of PKA C subunits in 293T treated without (-) or with (+) 340 μM 8-CPT-cAMP.

To test for differential roles of PKAI and PKAII expressed in one cell we tested if markedly different C subunits released from RI and RII are equally effective in regulating *in vivo *substrate phosphorylation. We chose the cell line 293T as a model system since they express RIα and RIIα associated with Cα1 (Figure 1A, left panel), and not RIβ and RIIβ (Figure 1A, right panel). In these cells PKAI and PKAII are distinctly located to the cytosol and Golgi-centrosomal area, respectively as demonstrated by immunostaining using anti-RIα (red) or anti-RIIα (green) (Figure 1B). Co-immunostaining with anti-C demonstrated that Cα1 localization corresponded to R subunit localization. We also observed a weak nuclear staining of the C subunit in the absence of cAMP (Figure 1C), whereas in the presence of the cAMP analogue, 8-CPT-cAMP (340 µM) an increase in nuclear staining was observed (Figure 1C). We concluded that the 293T cells represented a suitable model system to study isoform differences between PKAI and PKAII formed with different C subunits.

**Figure 2:**
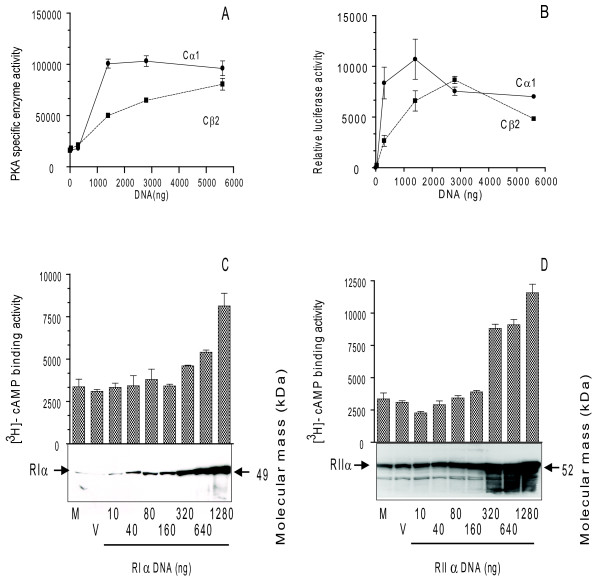
**Activity of PKA R and C subunits expressed in 293T cells**. **(A and B) **293T cells were left untransfected, transfected with empty vector (vector) or with increasing amounts (0 - 5600 ng) of either pDeCα1 (Cα1, —) or pDeCβ2 (Cβ2, ------). After 24 hours cells were harvested, homogenized and all cell extracts adjusted to 1 mg total protein/mL. PKA activity was determined as catalytic activity against Kemptide in the presence of 7.14 μM cAMP (**A**) and Luciferase activity at 560 nm (**B)**. Data points represent enzyme activity and relative luciferase activity, respectively, +/- SD, n = 3. (**C and D**) 293T cells were left untransfected, transfected with empty vector (vector) or with increasing amounts (10 - 1280 ng) of either pDeRIα or pExRIIα. Levels of R subunit expression were monitored as [^3^H]-cAMP-binding and R subunit immunoreactivity against RIα (**C**, clone 4D7, 1: 300 dilution) or anti-RIIα (**D**, 1 : 400 dilution) after SDS-PAGE separation of 25 μg total protein per lane in 12.5 *% *gels. R subunit activities are given as cpm +/- SD (n = 3). The apparent molecular weight of protein recognized is indicated by arrows and protein identity RIα (49 kDa) and RIIα (52 kDa), given by arrows to the left. One immunoblot out of three independent experiments is shown.

To obtain 293T cells dominated by either PKAI or PKAII expression, we formed holoenzymes by transient transfection of plasmids over-expressing either RIα or RIIα (pDeRIα or pExRIIα) in combination with either Cα1 or Cβ2 (pDeCα1 or pDeCβ2). For some experiments the cells were also transfected with a vector expressing Luciferace controlled by a cAMP responsive element. C subunit activity was tested *in vitro *using Kemptide as a substrate [43,44]; and *in vivo *using the Cre-Luciferase reporter system [45]. This revealed a dose-dependent increase in PKA-specific catalytic activity against Kemptide for both pDeCα1 and pDeCβ2 with a maximum at 5600 ng DNA (Figure 2A). The luciferase response was bell shaped and reached a maximum for pDeCα1 and pDeCβ2 at 1400 and 2800 ng DNA, respectively (Figure 2B). Next, we titrated the plasmids expressing RI and RII by transfecting 0-1280 ng of the plasmids pDeRIα and pExRIIα, respectively (Figure 2C, D).

Twenty four hours after transfection cells were lysed and R subunit levels were measured by immunoblotting and [^3^H]-cAMP-binding. This revealed an increase in a 49 kDa immunoreactive band as well as increased [^3^H]-cAMP-binding that coincided with the amount of plasmid transfected (pDeRIα, Figure 2C). The same was the case when transfecting pExRIIα (Figure 2D). Together this demonstrated a dose-dependent expression of both RIα and RIIα.

**Figure 3:**
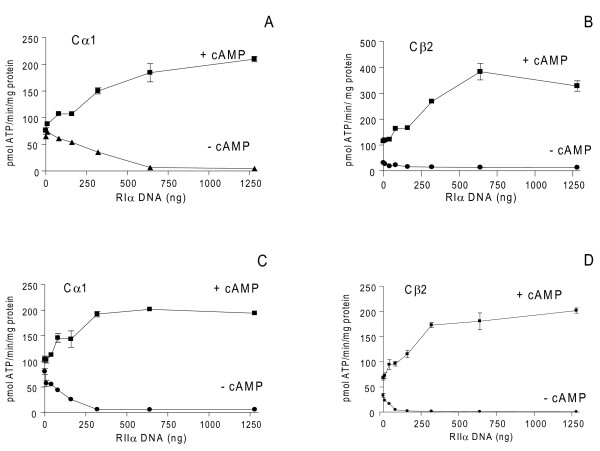
**Expressed RIα and RIIα inhibit expressed Cα1 and Cβ2 catalytic activity in a dose-dependent manner**. 293T cells were co-transfected with increasing amounts (0-1280 ng) of either pDeRIα (**A and B**) or pExRIIα (**C and D**) with a fixed amount of pDeCα1 (300 ng, **A and C**) or pDeCβ2 (1400 ng, **B and D**). Cells were harvested after 24 hours, cell extracts adjusted to 1 mg total protein/mL and assayed for PKA-specific phosphotransferase activity in the presence (+ cAMP) and absence (- cAMP) of 7.14 μM cAMP. Data points represent pmol ATP transferred/min/mg protein) +/- SD (n = 2-6).

**Figure 4:**
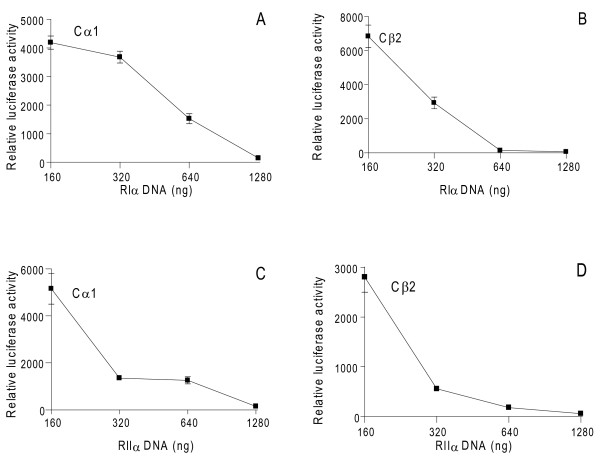
**Expressed RIα and RIIα inhibit Cα1- and Cβ2-dependent CREB phosphorylation in a dose-dependent manner**. 293T cells were co-transfected with increasing amounts (0-1280 ng) of either pDeRIα (**A and B**) or pExRIIα (**C and D**) and a fixed amount of pDeCα1 (300 ng, **A and C**) or pDeCβ2 (1400 ng, **B and D**). Cells were harvested after 24 hours and cell extracts adjusted to 1 mg total protein/mL and assayed at 560 nm for PKA-specific phosphorylation of CREB measured as CRE-activity. Data points represent relative luciferase activity +/- SD (n = 2-6).

Based on these transfections and earlier experiments (results not shown), we next formed PKA holoenzymes by R and C co-transfections. We aimed at transfecting R plasmids to levels where C activity in the absence of cAMP were at basal levels, implying levels of R able to associate with all C subunits. 293T cells were co-transfected with a fixed amount of either pDeCα1 (300 ng) or pDeCβ2 (1400 ng) together with increasing amounts of pDeRIα (0-1280 ng, Figure 3A, B) and pExRIIα (0-1280 ng, Figure 3C, D), respectively. Cell extracts were adjusted to 1 mg total protein/mL and total C subunit activity measured in the presence and absence of 7.14 μM cAMP. This demonstrated that Cα1-specific kinase activity was inhibited down to basal levels in the absence of cAMP at 640 ng pDeRIα (Figure 3A), which was equal to 28 ± 1.4 pmol RIα/mg total protein (Table 1). In the case of Cβ2-specific activity it was down to basal levels in the absence of cAMP at 80 ng pDeRIα (Figure 3B) which was equal to 11.8 ± 2.7 pmol RIα/mg total protein (Table 1). For RIIα, 320 ng pExRIIα was required for optimal Cα1 inhibition (Figure 3C), which was equal to 16.2 ± 0.5 pmol RIIα/mg total protein (Table 1). Finally, 80 ng pExRIIα was required to inhibit Cβ2 activity to basal levels (Figure 3D) which was equal to 9.6 ± 2 pmol RIIα/mg total protein. In order to compare *in vitro *and *in vivo *PKA activity, protein extracts were analyzed against Kemptide phosphorylation and luciferace activity after transfection with Cage-Cre-Luciferase (700 ng) together with either 300 ng pDeCα1 or 1400 ng pDeCβ2 and increasing amounts of pDeRIα and pExRIIα (160-1280 ng DNA, Figure 4A-D). In these experiments psv-β-Galactosidase (1000 ng) was used for normalization (see Methods). This showed that luciferase activity induced by Cα1 and Cβ2 was completely inhibited by both RIα and RIIα at doses above or equal to 640 ng plasmid DNA.

**Table 1:** Concentrations of RI and RII required for maximal inhibition of transfected C subunit

Subunits	Cα1 (300 ng DNA)	Cβ2 (1400 ng DNA)
RIα	28 ± 1.4 *	11.8 ± 2.7
RIIα	16.2 ± 0.5	9.6 ± 2

* Concentration of R subunit (pmol/mg protein) required for 100% inhibition of Cα1 and Cβ2 activity.

**Figure 5:**
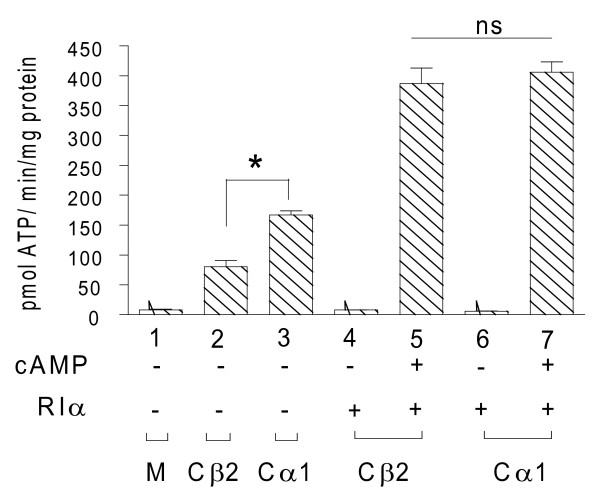
**Cα1 and Cβ2 proteins are stabilized by RIα**. 293T cells were untransfected (M) or transfected with fixed amounts of either pDeCα1 (300 ng) or pDeCβ2 (1400 ng) in conjunction with 1280 ng (bars 4 to 7) or without (bars 1-3) pDeRIα for 24 h. Cell extracts were adjusted to 1 mg total protein/mL and assayed for PKA-specific phosphotransferase activity in the absence (- cAMP) and presence (+ cAMP) of 320 nM cAMP. Data points represent pmol ATP transferred/min/mg protein) +/- SD (n = 3). The relative activities of Cβ2 and Cα1 in the absence of RIα were significantly different (* p < 0.02, bars 2 and 3). When Cα1 and Cβ2 were co-transfected with RIα the relative activities were indistinguishable (bars 5 and 7, ns).

The experiments in Figures 3 and 4 depict that Cβ2 activity is fully inhibited at lower amounts of R than Cα1 is. This may imply that Cα1 is enzymatically more active than Cβ2 or simply that Cβ2 is more unstable than Cα1 in the absence of R. A previous report shows that the C subunit in its free active form is more rapidly degraded than C complexed with the R subunit dimer [46]. To test if Cα1 and Cβ2 display differential stability, identical amounts of Cα1 and Cβ2 plasmids were transfected alone or with 1280 ng of pDeRIα. This confirmed (Figure 5 bars 2 and 3) that in the absence of RIα total Cβ2 activity is significantly (* p< 0.05) lower compared to Cα1. This was not the case when RIα was co-transfected with the two C subunits. In this case both Cα1 and Cβ2 activities were increased, however, to comparable levels after stimulation with cAMP (bars 5 and 7, ns). This demonstrated that RIα has a stabilizing effect on both C subunits. However the effect was more pronounced for Cβ2 than Cα1 indicating that Cβ2 is more unstable than Cα1 in the absence of R.

The results from Figures 3 and 4 demonstrated that we had obtained cell systems dominated by either PKAI or PKAII. Hence, the effects of PKAI and PKAII on *in vitro *(Kemptide) and *in vivo *(CREB) phosphorylation could be tested. For these experiments we used amounts of RIα and RIIα required for complete inhibition of Cα1 and Cβ2 respectively.

**Figure 6:**
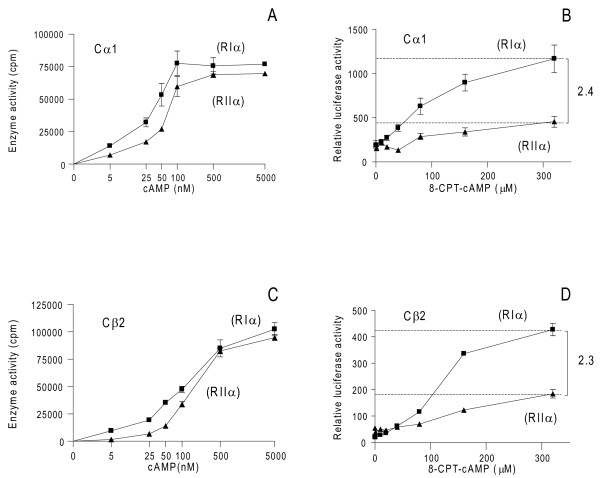
**Both PKAI and PKAII are activated to phosphorylate Kemptide but not CREB at saturating conditions of cAMP**. 293T cells were co-transfected with a fixed amount (1280 ng) of either pExRIIα or pDeRIα together with pDeCα1 (300 ng) (**A and B**) or pDeCβ2 (1400 ng) (**C and D**) for 24 hours. Cell extracts were adjusted to 1 mg total protein/mL and assayed for enzyme activity (cpm,) in the presence of increasing concentrations of cAMP (0 - 5000 nM) (**A and C**). Identical cell extracts were assayed for relative luciferase activity (**B and D**). Data points represent relative enzyme activity and luciferase activity, +/- SD (n = 3-6).

**Figure 7:**
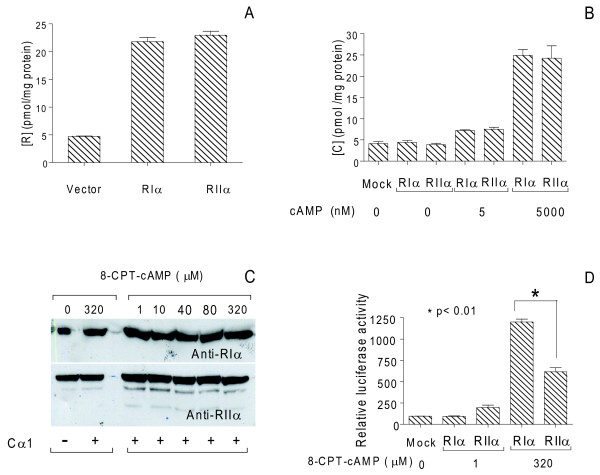
**PKAII is less efficient compared to PKAI in regulating CRE activity *in vivo***. 293T cells were co-transfected with or without (empty vector, M), pDeCα1 (300 ng) together with either pExRIIα (320 ng) or pDeRIα (640 ng) for 24 h (**A- C**). (**A**) R subunit-specific activity (pmol cAMP/mg protein) was monitored in the presence of 25 μM [^3^H]-cAMP and showed comparable levels of RIα and RIIα. (**B**) Catalytic activity of Cα1 was monitored in the absence (0) and presence of low (5 nM) and high (5000 nM) concentrations of cAMP. In the presence of 5000 nM cAMP levels of transfected Cα1 released from RIα and RIIα were equal. (**C**) The stability of RIα and RIIα was monitored in the presence (+) and absence (-) of Cα1 and the absence (0) and presence of incremental doses (1-320 μM) of 8-CPT-cAMP. R subunit stability was determined according to immunoreactive R subunits after separation of cell extracts (25 μg total protein) on SDS-PAGE (12.5 *% *gels) and anti-RIα (1 : 300 dilution, upper panel) and anti-RIIα (1 : 400 dilution, lower panel). (**D) **293T cells were left untreated (0) or stimulated for 1 hour with 8-CPT-cAMP (320 μM) before harvesting. Cell extracts were adjusted to 1 mg total protein/mL and assayed for PKA-specific phosphorylation of CREB monitored as relative luciferase activity at 560 nm. Data points represent relative luciferase activity +/- SD (n = 2-6).

After 24 hours cell extracts were diluted to 1 mg total protein/mL and analyzed for cAMP dose-dependent induction of PKA kinase activity against Kemptide (Figure 6A, C). Both RIα and RIIα were able to inhibit Cα1 and Cβ2 kinase activity completely in the absence of cAMP. When increasing the concentrations of cAMP from 5 to 5000 nM, kinase activity was peaking, in the case of Cα1 at 100 nM cAMP when co-expressed with RIα and between 500 and 5000 nM when co-expressed with RIIα. In the case of Cβ2, maximum activity was achieved at concentrations between 500 and 5000 nM cAMP when co-expressed with both RIα and RIIα. We further analyzed C subunit activity *in vivo *by measuring luciferace activity. Activity was measured after stimulation of the transfected cells with increasing concentrations of 8-CPT-cAMP (0 - 320 μM) for 1 hour prior to harvesting. We observed that activity associated with Cα1 and Cβ2 released from both RIα and RIIα increased in a dose-dependent manner, reaching maximum between 160 and 320 μM 8-CPT-cAMP (Figure 6B, D). However, a more than two fold higher activity was observed against CREB when Cα1 and Cβ2 were released from RIα than from RIIα. Together these results indicated that the ability of C to phosphorylate nuclear substrates *in vivo *at saturating concentrations of cAMP when associated with PKAII was lower than when associated with PKAI. This was apparent despite that total C subunit activity *in vitro *was comparable and protein concentrations were equal (Figure 6A to 6D). Since these results were seen regardless of C subunit isoform we suspected that the differences observed were associated with R subunit identity. To quantify the different efficacy of PKAI and PKAII to phosphorylate CREB *in vivo*, we therefore co-transfected pDeRIα (640 ng) and pExRIIα (320 ng) with Cα1 (300 ng pDeCα1) and monitored [^3^H]-cAMP binding. This showed equal activities (Figure 7A) and hence comparable levels (Table 2) revealed as 22 ± 1.5 and 23 ± 1.5 pmol per mg total protein of RIα and RIIα, respectively. We next determined C subunit activity *in vitro *after transfecting cells as described in Figure 7A, and in the absence (0 nM) and presence of two concentrations of cAMP (5 and 5000 nM). This revealed basal activity in the absence, and low level activity in the presence of 5 nM cAMP whereas 5000 nM cAMP resulted in comparable high levels of total C subunit activity released from both PKAI and PKAII (Figure 7B). The C activities were equal to 25 ± 1.4 and 24.2 ± 2.9 pmol Cα1 per mg total protein for PKAI and PKAII, respectively (Table 2). This concluded that PKAI and PKAII were expressed at comparable levels under the present conditions. The latter was substantiated by a calculated R to C ratio close to 1 for both RIα versus Cα1 (ratio 0.88) and RIIα versus Cα1 (ratio 0.96, Table 2).

**Table 2:** Ratios of transfected R and C subunits

PKA subunits	[R]*	[C]	R/C ratio
Holoenzyme			
PKAI	22 ± 1.5	25 ± 1.4	0.88
PKAII	23 ± 1.5	24 ± 2.9	0.96

* Concentration of R and C subunit (pmol/mg protein) were determined at saturating concentrations of cAMP and assuming two cAMP binding sites per R subunit and 600 pmol phosphate transferred by pure bovine C per min per mg [65].

In lymphoid cells, it has been demonstrated that R subunits are more stable in the holoenzyme form compared to the free R subunit [47]. To test if the presence of Cα1 alone and in conjunction with cAMP would influence R subunit levels we transfected 293T cells with either pDeRIα (640 ng) or pExRIIα (320 ng) alone or in conjunction with pDeCα1 (300 ng). Transfected cells were treated without (0) or with incremental doses (1-320 μM) of 8-CPT-cAMP for four hours before harvesting. Equal amounts of cell extracts (50 μg total protein per lane) were analyzed for proteins immunoreactive to anti-RIα and anti-RIIα, respectively. Figure 7C shows that 8-CPT-cAMP stimulation appeared not to influence R subunit levels and thus the cAMP sensitivity of the system.

Based on our observations (Figure 6B and 6D), we transfected cells as described in Figure 7B with equal amounts of PKAI and PKAII and monitored luciferace activity after stimulation with two concentrations of 8-CPT-cAMP (1 and 320 μM) for 1 hour before harvesting. As depicted in Figure 7D 320 μM 8-CPT-cAMP induced more than a 13-fold increase in luciferace activity when associated with RIα compared to untreated cells. When associated with RIIα the induction was 3-fold. This difference was reflected in a relative induction of luciferace activity which was nearly twice as high for PKAI compared to PKAII (1.94 fold, p < 0.01).

## Discussion

Despite that PKAI and PKAII are located to different areas when expressed in the same cell, it is believed that when dissociated by cAMP, the C subunits are all released to phosphorylate relevant substrates both in the cytosol and nucleus [48]. We formed PKAI and PKAII holoenzymes by co-transfecting 293T cells with RIα or RIIα together with either Cα1 or Cβ2.

We found that C subunits, irrespective of isoform, appeared more efficient in inducing Cre-luciferase when released from PKAI than PKAII.

To monitor total PKA activity *in vitro *and *in vivo *we applied cAMP and the cAMP analogue 8-CPT-cAMP. *In vitro *activation of PKA by cAMP was done to monitor if we had achieved comparable amounts of PKAI and PKAII in our experiments. For monitoring *in vivo *endogenous activity 8-CPT-cAMP was used because it has cell membrane permeable properties and is resistant to phosphodiesterase degradation [49]. The observation that cells transfected with PKAI induced higher levels of luciferace activity upon 8-CPT-cAMP stimulation than cells transfected with PKAII may have been due to relative affinities of the cAMP analogue. We consider this unlikely since 8-CPT-cAMP is a B-site selective cAMP analogue with higher affinity for RII than RI [49]. Further support for 8-CPT-cAMP as a competent activator of PKAII *in vivo *is found in that the concentration of 8-CPT-cAMP used is capable of displacing the C subunit from the RII subunit interacting with the centrosome *in vivo *in U2OS cells [50]. Taken together we conclude that 8-CPT-cAMP is fully capable to activate PKAII and does not selectively activate PKAI, implying that PKAII is less potent compared with PKAI in inducing Cre-luciferase activity.

An explanation for the biological significance of the phenomenon observed may rely on several factors. Despite that 25% of PKA is undissociated even in the presence of saturating concentrations of cAMP [51] it may not account for the differences we observed since this is observed for both PKAI and PKAII. However, it has been demonstrated that cAMP-dissociated RII and C reassociate much faster and to a much greater extent than RI and C. In fact it has been suggested that C does not really leave RII under physiological conditions due to a rapid reassociation [52]. Hence, incomplete dissociation of C subunit from RII even at saturating concentrations of cAMP could be a mechanism explaining the phenomena observed here. Moreover, the biological significance of differential effects of activating PKAI and PKAII independent of C subunit identity may be multiple. Recently a paper by Di and co-workers [53] demonstrated that PKAI and PKAII define distinct intracellular signaling compartments. They demonstrated that PKAI and PKAII activity were regulated by distinct, spatially restricted cAMP signals generated in response to specific G protein-coupled receptors and which were regulated by unique subsets of the cAMP degrading phosphodiesterases.

We observed that Cα1 was more active than Cβ2 when expressed in non-holoenzyme form. This may suggest differential K_d _of Cα1 and Cβ2 against RI and RII. This suggestion was supported in that the amount of R plasmid required for complete inhibition of Cα1 and Cβ2, respectively, was higher for RI compared to RII regardless of C subunit identity (28 pmol RIα/mg protein and 15 pmol RIIα/mg protein for Cα1 versus ~12 pmol RIα/mg protein and ~10 pmol RIIα/mg protein for Cβ2). However, we also observed that Cβ2, but not Cα1 activity was stabilized when co-transfecting with the R subunit implying that the differences observed is due to protein instability of the Cβ2 subunit and not lower K_d _for the R subunit.

The latter is supported by the observation that R and C dissociation by cAMP *in vivo *promotes degradation of C subunits through posttranslational mechanisms which may involve proteasome action [54]. Furthermore, it has been shown that Cα1 and Cβ1 have identical K_d _values for RI [55]. To what extent Cβ2 is more sensitive to proteasome degradation than Cα1 is not known. It should however be noted that the marked differences between the Cα1 and Cβ2 at the N-terminus has been implicated in C subunit stability. For Cα1 it has been demonstrated that the α-helix and Trp 30 are vital moieties for Cα1 stability. This correlates with the location of the N-terminal at the cleft interface where it orients the C-helix in the small lobe and the activation loop in the large lobe so that these subdomains are aligned in a way that allows for correct configuration of residues at the active site [56]. Moreover, we did not demonstrate a relative difference in potency of Cβ2 versus Cα1 in inducing Cre-luciferase activity irrespective of association with RIα or RIIα. The latter may suggest that Cα1 and Cβ2 behave identically in regulating Cre-luciferase activities. Hence, we concluded that the differential effects of PKAI and PKAII on luciferase activity detected in the present work are associated with R subunit but not C subunit. The latter was unexpected since it has been speculated if the marked sequence differences at the N-terminus will influence PKA holoenzyme features as such as localization. The latter has previously been demonstrated in that the N-terminus of Cα1 is implicated in subcellular anchoring to A-kinase interacting protein 1 (AKIP1) [57]. Furthermore, at the N-terminal end the myristoyl moiety, which binds to a hydrophobic pocket on the surface of the large lobe when Cα1 subunit is in the holoenzyme form [58,59], is exposed to the surroundings upon binding to RII. This makes the holoenzyme more hydrophobic [60]. In addition, whereas the N-terminal Asn moiety, is involved in fine-tuning of the enzyme distribution within the cell *in vivo *[61], Ser10 phosphorylation is known to introduce electro statically mediated forces which may help C to remain soluble even when myristoylated [62-64]. Together this implies the N-terminal of Cα1 to contribute to regulation and tuning of subcellular targeting. Despite lack of experimental evidence the N-terminal amphiphatic α-helix in Cβ2 has been proposed to function as a targeting domain for Cβ2 *in vivo *[20]. Despite the obvious differences between Cα1 and Cβ2 we did not observe any experimental evidence on the C subunits contributing to understand the differential effects of PKAI and PKAII.

In perspective, the various reports referred to here [51-53] together with our observations demonstrate differential activities and regulation by PKAI and PKAII which may add to understand the biological significance of two PKA holoenzymes expressed in one cell.

## Conclusions

This study is important because it points to how tissue-dependent expression of genes encoding subunits of PKA achieve specificity in the cAMP signaling pathway. Our work shows that transfected PKAI holoenzymes are more efficient than PKAII in phosphorylating CRE elements *in vivo *regardless of C subunit identity. Furthermore we show that Cβ2 appear more stable in the presence of R subunit than Cα1.

## Methods

### Cell culture

293T HEK cells were maintained in RPMI medium 1640 (Sigma) containing 10% (v/v) Fetal Bovine Serum (Sigma), 2 mM L-Glutamine (Sigma) 1*% *Non-essential amino acids (Gibco), 1*% *Na-Pyruvat (Gibco) and 1% (v/v) Penicillin/Streptomycin (Sigma). The cells were subcultured three times weekly. Twenty hours before transfection 293T cells were grown in 6 well plates from a population of 0.7 × 10^6 ^cells per well containing 1.5 mL RPMI medium without Penicillin/Streptomycin. Plates were kept at 37°C in a humidified atmosphere under 5*% *CO_2_.

### Generation and expression of PKA vectors

pEF-DEST 51™(Invitrogen) expression vectors encoding human regulatory and catalytic subunits RIα, Cα1 and Cβ2 were created using Gateway LR Clonase Reaction^® ^(Invitrogen) and transformed into Library^® ^efficiency DH5α™Competent cells (Invitrogen). Plasmid pBluescript containing RIIα encoding fragment was digested with Eag I (New England Biolabs), ligated using T4 Ligase (Promega) in plasmid pExchange 6A (Promega) previously digested with Not I (Promega), and transformed into Ultramax^® ^DH5α™Competent cells (Invitrogen). Plasmids expressing catalytic subunits Cα1 or Cβ2 or/and regulatory subunits RIIα or RIα herby termed pDeCα1, pDeCβ2, pDeRIα, and pExRIIα where transfected using Lipofectamine 2000 (Invitrogen). In order to facilitate a reporter system, plasmids expressing Luciferase reporter gene and β-Galactosidase as a normalization control was co-transfected with R subunit and/or C subunit in constant amounts (0.7 μg Cage-Cre-Luciferase reporter vector and 1 μg Psv-β-Galactosidase vector) in all wells except wells kept as "mock" controls. A vector without insert was used to keep the amount of plasmid DNA transfected constant. Cells were stimulated with 8-CPT-cAMP for 1 or 4 hours (specified in the text) before being harvested 24 hours post transfection.

### Immunoblot analysis

Immuno blotting was performed as previously described [15]. Membranes were incubated with mouse monoclonal anti-RIIβ (cat # 610625, BD Transduction laboratories) at 1:250 dilution, polyclonal rabbit anti-RIβ (cat # SC-907, Santa Cruz Biotechnology, Inc.), anti-RIIα (cat # 612243, BD Transduction laboratories) at 1:400 dilution or mouse monoclonal anti-RIα (Clone 4D7, [65]) at 1:300 dilution. Immunoreactive proteins were detected with HRP-conjugated secondary antibodies (ICN Diagnostics) and SuperSignal^® ^West Pico Chemiluminiscent (Pierce).

### Phosphotransferase assays

PKA-specific catalytic activity was determined as described previously [66]. Molar amounts of C subunit were determined assuming 600 pmol phosphate transferred per min per mg pure bovine C.

### Luciferase assay

Briefly, 24 hours post transfection cells were harvested, lysed by sonication, and samples adjusted to equal protein concentrations (1 mg/mL). Lysates were added appropriate buffer containing 270 μM Coenzyme A (Boehringer), 530 μM ATP (Boehringer), 470 μM Luciferin (SynChem), and immediately placed in a Luminometer (TD20/20, Turner Designs). Luminosity was measured after 2 seconds delay at 560 nm for 15 seconds with 20.1% of intensity. Samples in the high end of luminosity were used to create a standard curve to ensure measurement in the linear range.

### R-binding assay

The level of R-subunits was determined by specific [^3^H]-cAMP binding in homogenates from transfected 293T cells as previously described [15]. Molar amounts of R subunits were calculated assuming two cAMP binding sites per R subunit.

### Indirect Immunofluorescence (IF)

IF of 293T cells were performed as previously described [67]. Antibodies against RI (Clone 4D7, [65]) and RII (cat # 612243, BD Transduction laboratories) were diluted (see figure legend). The anti-C antibodies were rabbit polyclonal anti-Cα 1:100 (cat # sc 903, Santa Cruz Biotechnology, Santa Cruz, CA).

### Statistics

Data are presented as means ± s.e.m and were analyzed by unpaired two-tailed t test or by one-way analysis. A value of <0.05 was considered statistically significant. All statistics were calculated by the Graphpad prism 5.02 program.

## List of abbreviations

C: catalytic subunit; CREB: cAMP-responsive element binding protein; PKA: protein kinase A; R: regulatory subunit of PKA.

## Authors' contributions

ØS carried out most of the experiments, participated in the design of the study and in drafting the manuscript and preparing it for submission. ACVL participated in the experiments, provided technical assistance and contributed in criticizing the manuscript. AK performed indirect immunofluorescence experiments and contributed in criticizing the manuscript. SE participated in indirect immunofluorescence experiments and provided technical assistance. SØ conceived the design of the study, helped in its coordination and contributed in criticizing the manuscript. BSS conceived the design of the study, helped in its coordination and wrote the manuscript. All authors read and approved the final manuscript.

## References

[B1] Butcher RW Ho RJ Meng HC Sutherland EW Adenosine 3',5'-monophosphate in biological materials. II. The measurement of adenosine 3',5'-monophosphate in tissues and the role of the cyclic nucleotide in the lipolytic response of fat to epinephrine J Biol Chem 1965 240 4515 4523 4378937

[B2] Walsh DA Perkins JP Krebs EG An adenosine 3',5'-monophosphate-dependant protein kinase from rabbit skeletal muscle J Biol Chem 1968 243 3763 3765 4298072

[B3] Doskeland SO Evidence that rabbit muscle protein kinase has two kinetically distinct binding sites for adenosine 3'; 5'-cyclic monophosphate Biochem Biophys Res Commun 1978 83 542 549 10.1016/0006-291X(78)91024-0 212058

[B4] Corbin JD Keely SL Park CR The distribution and dissociation of cyclic adenosine 3':5'-monophosphate-dependent protein kinases in adipose, cardiac, and other tissues J Biol Chem 1975 250 218 225 166986

[B5] Reimann EM Brostrom CO Corbin JD King CA Krebs EG Separation of regulatory and catalytic subunits of the cyclic 3',5'-adenosine monophosphate-dependent protein kinase(s) of rabbit skeletal muscle Biochem Biophys Res Commun 1971 42 187 194 10.1016/0006-291X(71)90086-6 4322813

[B6] Maller JL Kemp BE Krebs EG In vivo phosphorylation of a synthetic peptide substrate of cyclic AMP-dependent protein kinase Proc Natl Acad Sci USA 1978 75 248 251 10.1073/pnas.75.1.248 203933 PMC411223

[B7] Montminy MR Bilezikjian LM Binding of a nuclear protein to the cyclic-AMP response element of the somatostatin gene Nature 1987 328 175 178 10.1038/328175a0 2885756

[B8] Yamamoto KK Gonzalez GA Biggs WH Montminy MR Phosphorylation-induced binding and transcriptional efficacy of nuclear factor CREB Nature 1988 334 494 498 10.1038/334494a0 2900470

[B9] Skalhegg BS Tasken K Specificity in the cAMP/PKA signaling pathway. Differential expression, regulation, and subcellular localization of subunits of PKA Front Biosci 2000 5 D678 D693 10.2741/Skalhegg 10922298

[B10] Solberg R Sandberg M Natarajan V Torjesen PA Hansson V Jahnsen T Tasken K The human gene for the regulatory subunit RI alpha of cyclic adenosine 3', 5'-monophosphate-dependent protein kinase: two distinct promoters provide differential regulation of alternately spliced messenger ribonucleic acids Endocrinology 1997 138 169 181 10.1210/en.138.1.169 8977401

[B11] Barradeau S Imaizumi-Scherrer T Weiss MC Faust DM Alternative 5'-exons of the mouse cAMP-dependent protein kinase subunit RIalpha gene are conserved and expressed in both a ubiquitous and tissue-restricted fashion FEBS Lett 2000 476 272 276 10.1016/S0014-5793(00)01653-7 10913627

[B12] Dahle MK Knutsen HK Tasken KA Pilz R Tasken K Cyclic AMP regulates expression of the RI alpha subunit of cAMP-dependent protein kinase through an alternatively spliced 5' UTR Eur J Biochem 2001 268 5920 5929 10.1046/j.0014-2956.2001.02542.x 11722580

[B13] Johansson CC Dahle MK Blomqvist SR Gronning LM Aandahl EM Enerback S Tasken K A winged helix forkhead (FOXD2) tunes sensitivity to cAMP in T lymphocytes through regulation of cAMP-dependent protein kinase RIalpha J Biol Chem 2003 278 17573 17579 10.1074/jbc.M300311200 12621056

[B14] Oyen O Myklebust F Scott JD Hansson V Jahnsen T Human testis cDNA for the regulatory subunit RII alpha of cAMP-dependent protein kinase encodes an alternate amino-terminal region FEBS Lett 1989 246 57 64 10.1016/0014-5793(89)80253-4 2540040

[B15] Kvissel AK Orstavik S Oistad P Rootwelt T Jahnsen T Skalhegg BS Induction of Cbeta splice variants and formation of novel forms of protein kinase A type II holoenzymes during retinoic acid-induced differentiation of human NT2 cells Cell Signal 2004 16 577 587 10.1016/j.cellsig.2003.08.014 14751543

[B16] Orstavik S Reinton N Frengen E Langeland BT Jahnsen T Skalhegg BS Identification of novel splice variants of the human catalytic subunit Cbeta of cAMP-dependent protein kinase Eur J Biochem 2001 268 5066 5073 10.1046/j.0014-2956.2001.02429.x 11589697

[B17] Reinton N Orstavik S Haugen TB Jahnsen T Tasken K Skalhegg BS A novel isoform of human cyclic 3',5'-adenosine monophosphate-dependent protein kinase, c alpha-s, localizes to sperm midpiece Biol Reprod 2000 63 607 611 10.1095/biolreprod63.2.607 10906071

[B18] Showers MO Maurer RA Cloning of cDNA for the catalytic subunit of cAMP-dependent protein kinase Methods Enzymol 1988 159 311 318 full_text 3412179 10.1016/0076-6879(88)59031-6

[B19] Uhler MD Carmichael DF Lee DC Chrivia JC Krebs EG McKnight GS Isolation of cDNA clones coding for the catalytic subunit of mouse cAMP-dependent protein kinase Proc Natl Acad Sci USA 1986 83 1300 1304 10.1073/pnas.83.5.1300 3456589 PMC323063

[B20] Wiemann S Kinzel V Pyerin W Isoform C beta 2, an unusual form of the bovine catalytic subunit of cAMP-dependent protein kinase J Biol Chem 1991 266 5140 5146 2002051

[B21] Desseyn JL Burton KA McKnight GS Expression of a nonmyristylated variant of the catalytic subunit of protein kinase A during male germ-cell development Proc Natl Acad Sci USA 2000 97 6433 6438 10.1073/pnas.97.12.6433 10841548 PMC18620

[B22] Guthrie CR Skalhegg BS McKnight GS Two novel brain-specific splice variants of the murine Cbeta gene of cAMP-dependent protein kinase J Biol Chem 1997 272 29560 29565 10.1074/jbc.272.47.29560 9368018

[B23] Carr SA Biemann K Shoji S Parmelee DC Titani K n-Tetradecanoyl is the NH2-terminal blocking group of the catalytic subunit of cyclic AMP-dependent protein kinase from bovine cardiac muscle Proc Natl Acad Sci USA 1982 79 6128 6131 10.1073/pnas.79.20.6128 6959104 PMC347072

[B24] Jedrzejewski PT Girod A Tholey A Konig N Thullner S Kinzel V Bossemeyer D A conserved deamidation site at Asn 2 in the catalytic subunit of mammalian cAMP-dependent protein kinase detected by capillary LC-MS and tandem mass spectrometry Protein Sci 1998 7 457 469 10.1002/pro.5560070227 9521123 PMC2143929

[B25] Herberg FW Bell SM Taylor SS Expression of the catalytic subunit of cAMP-dependent protein kinase in Escherichia coli: multiple isozymes reflect different phosphorylation states Protein Eng 1993 6 771 777 10.1093/protein/6.7.771 8248101

[B26] Toner-Webb J van Patten SM Walsh DA Taylor SS Autophosphorylation of the catalytic subunit of cAMP-dependent protein kinase J Biol Chem 1992 267 25174 25180 1460017

[B27] Yonemoto W Garrod SM Bell SM Taylor SS Identification of phosphorylation sites in the recombinant catalytic subunit of cAMP-dependent protein kinase J Biol Chem 1993 268 18626 18632 8395513

[B28] San Agustin JT Leszyk JD Nuwaysir LM Witman GB The catalytic subunit of the cAMP-dependent protein kinase of ovine sperm flagella has a unique amino-terminal sequence J Biol Chem 1998 273 24874 24883 10.1074/jbc.273.38.24874 9733793

[B29] Funderud A Henanger HH Hafte TT Amieux PS Orstavik S Skalhegg BS Identification, cloning and characterization of a novel 47 kDa murine PKA C subunit homologous to human and bovine Cbeta2 BMC Biochem 2006 7 20 10.1186/1471-2091-7-20 16889664 PMC1557514

[B30] Orstavik S Funderud A Hafte TT Eikvar S Jahnsen T Skalhegg BS Identification and characterization of novel PKA holoenzymes in human T lymphocytes FEBS J 2005 272 1559 1567 10.1111/j.1742-4658.2005.04568.x 15794744

[B31] Fossberg TM Doskeland SO Ueland PM Protein kinases in human renal cell carcinoma and renal cortex. A comparison of isozyme distribution and of responsiveness to adenosine 3':5'-cyclic monophosphate Arch Biochem Biophys 1978 189 272 281 10.1016/0003-9861(78)90224-2 213022

[B32] Goel S Desai K Bulgaru A Fields A Goldberg G Agrawal S Martin R Grindel M Mani S A safety study of a mixed-backbone oligonucleotide (GEM231) targeting the type I regulatory subunit alpha of protein kinase A using a continuous infusion schedule in patients with refractory solid tumors Clin Cancer Res 2003 9 4069 4076 14519628

[B33] Bradbury AW Carter DC Miller WR Cho-Chung YS Clair T Protein kinase A (PK-A) regulatory subunit expression in colorectal cancer and related mucosa Br J Cancer 1994 69 738 742 10.1038/bjc.1994.139 8142263 PMC1968829

[B34] McDaid HM Cairns MT Atkinson RJ McAleer S Harkin DP Gilmore P Johnston PG Increased expression of the RIalpha subunit of the cAMP-dependent protein kinase A is associated with advanced stage ovarian cancer Br J Cancer 1999 79 933 939 10.1038/sj.bjc.6690149 10070893 PMC2362667

[B35] Amieux PS McKnight GS The essential role of RI alpha in the maintenance of regulated PKA activity Ann N Y Acad Sci 2002 968 75 95 10.1111/j.1749-6632.2002.tb04328.x 12119269

[B36] Kirschner LS Kusewitt DF Matyakhina L Towns WH Carney JA Westphal H Stratakis CA A mouse model for the Carney complex tumor syndrome develops neoplasia in cyclic AMP-responsive tissues Cancer Res 2005 65 4506 4514 10.1158/0008-5472.CAN-05-0580 15930266

[B37] Kirschner LS Yin Z Jones GN Mahoney E Mouse models of altered protein kinase A signaling Endocr Relat Cancer 2009 16 773 793 10.1677/ERC-09-0068 19470615

[B38] Erlichman J Rosenfeld R Rosen OM Phosphorylation of a cyclic adenosine 3':5'-monophosphate-dependent protein kinase from bovine cardiac muscle J Biol Chem 1974 249 5000 5003 4367815

[B39] Wu J Brown SH von DS Taylor SS PKA type IIalpha holoenzyme reveals a combinatorial strategy for isoform diversity Science 2007 318 274 279 10.1126/science.1146447 17932298 PMC4036697

[B40] Beene DL Scott JD A-kinase anchoring proteins take shape Curr Opin Cell Biol 2007 19 192 198 10.1016/j.ceb.2007.02.011 17317140 PMC3521038

[B41] Tasken K Aandahl EM Localized effects of cAMP mediated by distinct routes of protein kinase A Physiol Rev 2004 84 137 167 10.1152/physrev.00021.2003 14715913

[B42] Manni S Mauban JH Ward CW Bond M Phosphorylation of the cAMP-dependent protein kinase (PKA) regulatory subunit modulates PKA-AKAP interaction, substrate phosphorylation, and calcium signaling in cardiac cells J Biol Chem 2008 283 24145 24154 10.1074/jbc.M802278200 18550536 PMC2527120

[B43] Witt JJ Roskoski R Jr Rapid protein kinase assay using phosphocellulose-paper absorption Anal Biochem 1975 66 253 258 10.1016/0003-2697(75)90743-5 1147218

[B44] Kemp BE Graves DJ Benjamini E Krebs EG Role of multiple basic residues in determining the substrate specificity of cyclic AMP-dependent protein kinase J Biol Chem 1977 252 4888 4894 194899

[B45] Mellon PL Clegg CH Correll LA McKnight GS Regulation of transcription by cyclic AMP-dependent protein kinase Proc Natl Acad Sci USA 1989 86 4887 4891 10.1073/pnas.86.13.4887 2544878 PMC297520

[B46] Alhanaty E Shaltiel S Limited proteolysis of the catalytic subunit of cAMP-dependent protein kinase--a membranal regulatory device? Biochem Biophys Res Commun 1979 89 323 332 10.1016/0006-291X(79)90633-8 226083

[B47] Skalhegg BS Johansen AK Levy FO Andersson KB Aandahl EM Blomhoff HK Hansson V Tasken K Isozymes of cyclic AMP-dependent protein kinases (PKA) in human lymphoid cell lines: levels of endogenous cAMP influence levels of PKA subunits and growth in lymphoid cell lines J Cell Physiol 1998 177 85 93 10.1002/(SICI)1097-4652(199810)177:1<85::AID-JCP9>3.0.CO;2-A 9731748

[B48] Harootunian AT Adams SR Wen W Meinkoth JL Taylor SS Tsien RY Movement of the free catalytic subunit of cAMP-dependent protein kinase into and out of the nucleus can be explained by diffusion Mol Biol Cell 1993 4 993 1002 8298196 10.1091/mbc.4.10.993 PMC275733

[B49] Genieser HG Winkler E Butt E Zorn M Schulz S Iwitzki F Stormann R Jastorff B Doskeland SO Ogreid D Derivatives of 1-beta-D-ribofuranosylbenzimidazole 3',5'-phosphate that mimic the actions of adenosine 3',5'-phosphate (cAMP) and guanosine 3',5'-phosphate (cGMP) Carbohydr Res 1992 234 217 235 10.1016/0008-6215(92)85050-A 1334800

[B50] Kvissel AK Orstavik S Eikvar S Brede G Jahnsen T Collas P Akusjarvi G Skalhegg BS Involvement of the catalytic subunit of protein kinase A and of HA95 in pre-mRNA splicing Exp Cell Res 2007 313 2795 2809 10.1016/j.yexcr.2007.05.014 17594903

[B51] Kopperud R Christensen AE Kjarland E Viste K Kleivdal H Doskeland SO Formation of inactive cAMP-saturated holoenzyme of cAMP-dependent protein kinase under physiological conditions J Biol Chem 2002 277 13443 13448 10.1074/jbc.M109869200 11834733

[B52] Viste K Kopperud RK Christensen AE Doskeland SO Substrate enhances the sensitivity of type I protein kinase a to cAMP J Biol Chem 2005 280 13279 13284 10.1074/jbc.M413065200 15691833

[B53] Di BG Zoccarato A Lissandron V Terrin A Li X Houslay MD Baillie GS Zaccolo M Protein kinase A type I and type II define distinct intracellular signaling compartments Circ Res 2008 103 836 844 10.1161/CIRCRESAHA.108.174813 18757829

[B54] Boundy VA Chen J Nestler EJ Regulation of cAMP-dependent protein kinase subunit expression in CATH.a and SH-SY5Y cells J Pharmacol Exp Ther 1998 286 1058 1065 9694969

[B55] Cadd GG Uhler MD McKnight GS Holoenzymes of cAMP-dependent protein kinase containing the neural form of type I regulatory subunit have an increased sensitivity to cyclic nucleotides J Biol Chem 1990 265 19502 19506 2174040

[B56] Herberg FW Zimmermann B McGlone M Taylor SS Importance of the A-helix of the catalytic subunit of cAMP-dependent protein kinase for stability and for orienting subdomains at the cleft interface Protein Sci 1997 6 569 579 10.1002/pro.5560060306 9070439 PMC2143671

[B57] Sastri M Barraclough DM Carmichael PT Taylor SS A-kinase-interacting protein localizes protein kinase A in the nucleus Proc Natl Acad Sci USA 2005 102 349 354 10.1073/pnas.0408608102 15630084 PMC544310

[B58] Bossemeyer D Engh RA Kinzel V Ponstingl H Huber R Phosphotransferase and substrate binding mechanism of the cAMP-dependent protein kinase catalytic subunit from porcine heart as deduced from the 2.0 A structure of the complex with Mn2+ adenylyl imidodiphosphate and inhibitor peptide PKI(5-24) EMBO J 1993 12 849 859 8384554 10.1002/j.1460-2075.1993.tb05725.x PMC413283

[B59] Zheng J Knighton DR Xuong NH Taylor SS Sowadski JM Ten Eyck LF Crystal structures of the myristylated catalytic subunit of cAMP-dependent protein kinase reveal open and closed conformations Protein Sci 1993 2 1559 1573 10.1002/pro.5560021003 8251932 PMC2142252

[B60] Gangal M Clifford T Deich J Cheng X Taylor SS Johnson DA Mobilization of the A-kinase N-myristate through an isoform-specific intermolecular switch Proc Natl Acad Sci USA 1999 96 12394 12399 10.1073/pnas.96.22.12394 10535933 PMC22929

[B61] Pepperkok R Hotz-Wagenblatt A Konig N Girod A Bossemeyer D Kinzel V Intracellular distribution of mammalian protein kinase A catalytic subunit altered by conserved Asn2 deamidation J Cell Biol 2000 148 715 726 10.1083/jcb.148.4.715 10684253 PMC2169370

[B62] Hanakam F Gerisch G Lotz S Alt T Seelig A Binding of hisactophilin I and II to lipid membranes is controlled by a pH-dependent myristoyl-histidine switch Biochemistry 1996 35 11036 11044 10.1021/bi960789j 8780505

[B63] Hanakam F Albrecht R Eckerskorn C Matzner M Gerisch G Myristoylated and non-myristoylated forms of the pH sensor protein hisactophilin II: intracellular shuttling to plasma membrane and nucleus monitored in real time by a fusion with green fluorescent protein EMBO J 1996 15 2935 2943 8670794 PMC450234

[B64] McLaughlin S Aderem A The myristoyl-electrostatic switch: a modulator of reversible protein-membrane interactions Trends Biochem Sci 1995 20 272 276 10.1016/S0968-0004(00)89042-8 7667880

[B65] Skalhegg BS Landmark BF Doskeland SO Hansson V Lea T Jahnsen T Cyclic AMP-dependent protein kinase type I mediates the inhibitory effects of 3',5'-cyclic adenosine monophosphate on cell replication in human T lymphocytes J Biol Chem 1992 267 15707 15714 1379235

[B66] Larsen ACV Kvissel AK Hafte TT Avellan CIA Eikvar S Rootwelt T Orstavik S Skalhegg BS Inactive forms of the catalytic subunit of protein kinase A are expressed in the brain of higher primates Febs Journal 2008 275 250 262 10.1111/j.1742-4658.2007.06195.x 18070107

[B67] Kvissel AK Orstavik S Eikvar S Brede G Jahnsen T Collas P Akusjarvi G Skalhegg BS Involvement of the catalytic subunit of protein kinase A and of HA95 in pre-mRNA splicing Exp Cell Res 2007 313 2795 2809 10.1016/j.yexcr.2007.05.014 17594903

